# Effects of low-dose X-ray irradiation on melanin-derived radicals in mouse hair and skin

**DOI:** 10.3164/jcbn.20-34

**Published:** 2020-06-03

**Authors:** Ken-ichiro Matsumoto, Megumi Ueno, Ikuo Nakanishi, Hiroko P. Indo, Hideyuki J. Majima

**Affiliations:** 1 Quantitative RedOx Sensing Group, Department of Basic Medical Sciences for Radiation Damages, National Institute of Radiological Sciences, Quantum Medical Science Directorate, 4-9-1 Anagawa, Inage-ku, Chiba 263-8555, Japan; 2 Quantum-state Controlled MRI Group, Institute for Quantum Life Science, National Institutes for Quantum and Radiological Science and Technology, 4-9-1 Anagawa, Inage-ku, Chiba 263-8555, Japan; 3 Department of Oncology, Graduate School of Medical and Dental Sciences, Kagoshima University, Kagoshima 890-8544, Japan; 4 Amanogawa Galactic Astronomy Research Center, Graduate School of Medical and Dental Sciences, Kagoshima University, Kagoshima 890-8544, Japan; 5 Department of Space Environmental Medicine, Graduate School of Medical and Dental Sciences, Kagoshima University, Kagoshima 890-8544, Japan

**Keywords:** low-dose irradiation, melanin-derived radicals, electron paramagnetic resonance, hair, skin

## Abstract

To clarify a possible index for long-term and low-dose irradiation, the effects of repeated low-dose X-ray irradiation on the amount of melanin-derived radicals in mouse hair and tail skin were investigated. Eight-week-old female C3H/HeSlc mice were irradiated by X-rays at a dose of 100 mGy/day 5 days/week for 12 weeks. Similarly, a 4-week irradiation experiment was carried out at 500 mGy/day for C3H/HeSlc mice, or at 10, 100, and 500 mGy/day for 8-week-old female C57BL/6NCrSlc mice. The hair sample (~10 mg) was weighed accurately and stuffed into a plastic tube. The 2-cm tip of the tail was sampled and lyophilized. Melanin-derived radicals in hair and tail samples were measured by X-band electron paramagnetic resonance spectrometry. After X-ray irradiation at 100 mGy/day for 12 weeks, no difference was found in the amount of melanin-derived radicals in the hair of the irradiated and non-irradiated groups. X-ray irradiation at 500 mGy/day for 4 weeks increased the amount of melanin-derived radicals in hair compared with the non-irradiated group, but the baseline amount of melanin-derived radicals in hair was varied. The amount of melanin-derived radicals in the tail skin dose-dependently increased. Melanin-derived radicals in skin may be an endogenous marker for long-term and low-dose irradiation.

## Introduction

All living organisms on Earth are exposed to natural radiation. In addition, humans may be exposed to artificial radiation such as medical exposure and/or occupational exposure. Patients receive medical exposure from clinical examinations using ionizing radiation, such as X-ray photograms, X-ray CT, positron emission tomography (PET), and/or single-photon emission computed tomography (SPECT). For example, the dose of a dental X-ray photogram is 2–10 µSv/examination, that of a chest X-ray photogram is 0.06 mSv/examination, for chest X-ray CT, the dose is 5–30 mSv/examination, and for PET, the dose is 2–20 mSv/examination.^(1)^ Other typical medical exposure consists of radiation therapy, the dose of which is over 60 Gy to the target region, i.e., tumor/cancer. The dose of cosmic radiation to an astronaut working on the International Space Station has been estimated to be 20.8–41.6 µSv/h,^(2)^ which is almost 100–200-times higher than the average dose to a person on the ground in Japan (2.4 mSv/year). However, estimation of the biological effects of such low-dose irradiation is difficult because almost no marked symptoms or death can be observed in an individual animal/human due to the dose of irradiation in space. Internal and/or external sensitive markers are therefore required to estimate the effects of low-dose irradiation.

Melanin, a black or brownish pigment, is not a single compound, but is a class of polymer compounds assembled by several chemical structural components. Melanin contains endogenous free radicals, termed intrinsic melanin-derived radicals, which are stabilized on the complicated polymer structure. The intrinsic melanin-derived radicals are a class of plural free radical components that can be directly measured using an electron paramagnetic resonance (EPR) spectrometer at room temperature.^(3,4)^

Melanin-derived radicals are the only endogenous free radical species noninvasively measured at room temperature. Ogawa *et al.*^(5)^ detected the intrinsic melanin-derived radicals in the living mouse tail non-invasively using an X-band EPR spectrometer, and reported increasing reactions to repeated daily 2-Gy irradiation. Diagnostic procedures for melanomas have been under development using EPR spectroscopy/imaging and/or DNP imaging techniques, as they can directly detect and visualize melanin-derived radicals in melanomas.^(6–8)^

The purpose of this study was to evaluate the possibility of melanin-derived radicals in hair and/or skin as an easily collectable sample for assessing long-term low-dose radiobiological effects. To find a possible index for clinical low-dose irradiation, the effects of repeated low-dose whole body X-ray irradiation on the amount of melanin-derived radicals in mouse back hair or tail skin was investigated. In addition, the effects of an antioxidant, 4-hydroxyl-2,2,6,6-tetramethylpiperidine-*N*-oxyl (TPL), on the melanin-derived radicals were detected as the relationship between reactive oxygen species (ROS) and the generation of melanin-derived radicals.

## Materials and Methods

### Chemicals

TPL was purchased from Sigma-Aldrich (St. Louis, MO). Deionized water (deionization by the Milli-Q system) was used as drinking water for mice.

### Animals

Seven-week-old female C3H/HeSlc or C57BL/6NCrSlc mice were purchased from Japan SLC, Inc. (Hamamatsu, Shizuoka, Japan). Mice were housed and habituated for a week, and used for experiments. Mice were housed three per cage. Mice were housed in a room with a 12-h light/dark cycle, and allowed food and water *ad libitum* during the experiment. Experiments were carried out in compliance with the Guidelines for Animal Experiments of the National Institute of Radiological Sciences.

### X-ray irradiation

Repetitive fractionated X-ray irradiation of healthy female mice was started from 8 weeks of age. Whole-body X-ray irradiation was performed on conscious mice. Irradiation protocols are described below.

Protocol 1: C3H/HeSlc mice were irradiated at 0 or 100 mGy a day, 5 times a week (Monday to Friday) for 12 weeks. The total dose was 0 or 6 Gy, respectively. The dose rate during irradiation was 200 mGy/min. The mice were kept in cages supplying drinking water with or without 1 mM TPL for the 12-week period of fractionated irradiation.

Protocol 2: C3H/HeSlc mice were irradiated at 0 or 500 mGy a day, 5 time a week for 4 weeks. The total dose was 0 or 10 Gy, respectively. The dose rate irradiation was 200 mGy/min. The mice were kept in cages supplying drinking water with or without 1 mM TPL for 4 weeks.

Protocol 3: C57BL/6NCrSlc mice were irradiated at 0, 10, 100, or 500 mGy a day, 5 times a week for 4 weeks. The total dose was 0.2, 2.0, or 10 Gy, respectively. The mice were kept in cages supplying Milli-Q water for 4 weeks.

X-ray irradiation was performed using PANTAK-320S (Shimadzu, Kyoto, Japan). The effective energy was 80 keV under the following conditions: X-ray tube voltage was 200 kV, X-ray tube current was 20 mA, and the thickness and materials of the pre-filter were 0.5-mm copper and 0.5-mm aluminum. The dose rate of X-ray irradiation for mouse tails was 0.24 Gy/min when the distance between the X-ray tube and the tail was 1,000 mm.

### Preparation of hair and tail samples

One hour before starting irradiation, the back hair of each mouse was shaved by electric hair clippers. An aliquot of hair (5–10 mg) was stuffed into a plastic tube (inner diameter: 1.4 mm, outer diameter: 2.3 mm, length: 20 mm) and weighed accurately. Similarly, after the series of fractionated X-ray irradiation, the newly grown back hair of the mice was again shaved and stuffed into the plastic tube. The hair samples were measured using an EPR spectrometer. The mice were sacrificed by cervical dislocation under anesthesia, and then the 2-cm tip of the tail was collected from each mouse. The tail tip samples were lyophilized and used for EPR measurement.

### *In vitro* EPR measurement of melanin-derived radicals

The hair stuffed into plastic tubes was placed in a quartz EPR sample holder and fixed in the TE-mode EPR cavity. The sample was measured using an X-band EPR spectrometer (JEOL, Tokyo). EPR conditions were as follows: microwave frequency, 9.4 GHz; microwave power, 2.00 mW; field modulation frequency, 100 kHz; field modulation amplitude, 0.063 mT; time constant, 0.3 s; and magnetic field sweep rate, 7.5 mT/min. The relatively broad single peak of melanin-derived radicals was measured by scanning one-fourth of the lower magnetic field of ±7.5-mT sweep width at 342.4 mT as the center magnetic field. EPR data acquisition was controlled by the WIN-RAD ESR Data Analyzer System (Radical Research, Inc., Hino, Tokyo, Japan). The acquired EPR spectrum was analyzed using an in-house line fitting program, and a 100% Gaussian line shape was fitted. The signal height and the line width of the fitted Gaussian line were measured. The EPR signal intensity was calculated as (signal height) × (linewidth)^2^.

### Statistical analysis

Statistical differences were estimated using the TTEST function in Microsoft Excel 2010. Suitable ‘tail’ and ‘type’ for the TTEST function were selected as follows. The ‘tail’ was 2 (two-tailed distribution) for stability tests because the difference between the two data groups was compared simply. The ‘type’ was selected according to the equality of variances between the data sets, which was estimated using the FTEST function. Grades of significance were estimated by *p*<0.05, *p*<0.01, and *p*<0.001.

## Results and Discussion

**Fig. 1:**
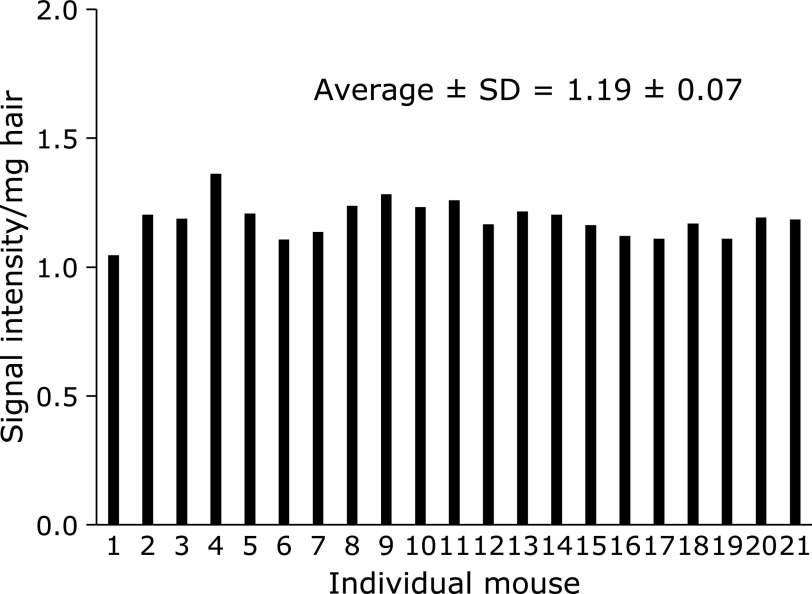
Individual variability in hair melanin-derived radicals in mouse back hair. Each column indicates an EPR signal intensity value, which was standardized by the weight of the hair sample measured. Each hair sample was prepared for an individual mouse. The average ± SD (a.u.) of the EPR signal intensity was 1.19 ± 0.07 (*n* = 21).

The individual variability of EPR signal intensity observed for melanin-derived radicals in back hair of non-irradiated mice is shown in Fig. 1. EPR signals of the intrinsic melanin-derived radicals in the hair were observed for all non-irradiated mice. Each column in Fig. 1 indicates an EPR signal intensity value, which was standardized by the weight of the hair sample measured. Preparation of the hair sample and measurement were performed for the 21 individual mice. The variation, i.e., SD value, of EPR measurements of melanin-derived radicals for non-irradiated control mice was 5.9% of the average value.

**Fig. 2:**
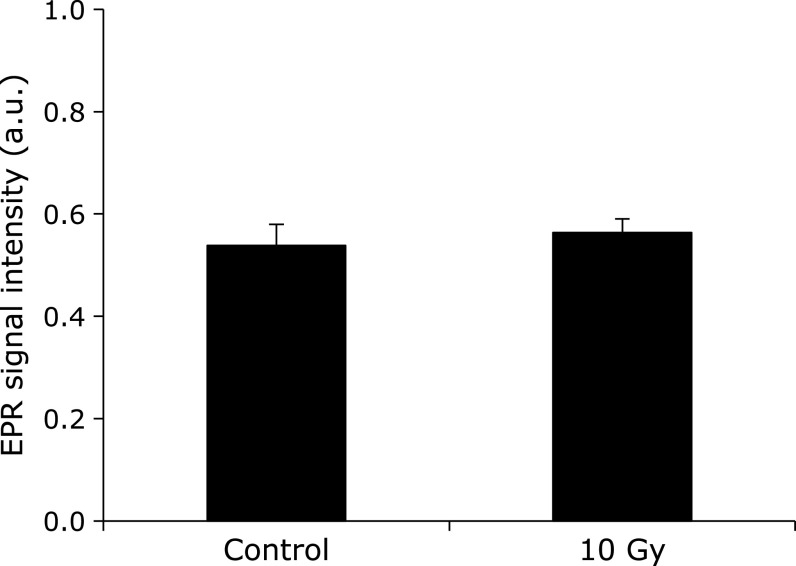
Effects of X-ray irradiation on the shaved hair sample. The columns and errors bar indicate the average ± SD of 3 mice. No significance was observed.

No changes were noted in the amount of melanin-derived radicals in the hair sample after being irradiated by a single dose of 10 Gy of X-rays (Fig. 2). Ogawa *et al.*^(5)^ also reported that the amount of melanin-derived radicals in commercial melanin powder did not change even after a single 20-Gy dose of X-ray irradiation. This suggests that the generation of stable melanin-derived radicals requires *in vivo* redox reactions. Direct ionization of the melanin molecule may not lead to stable radicals.

**Fig. 3:**
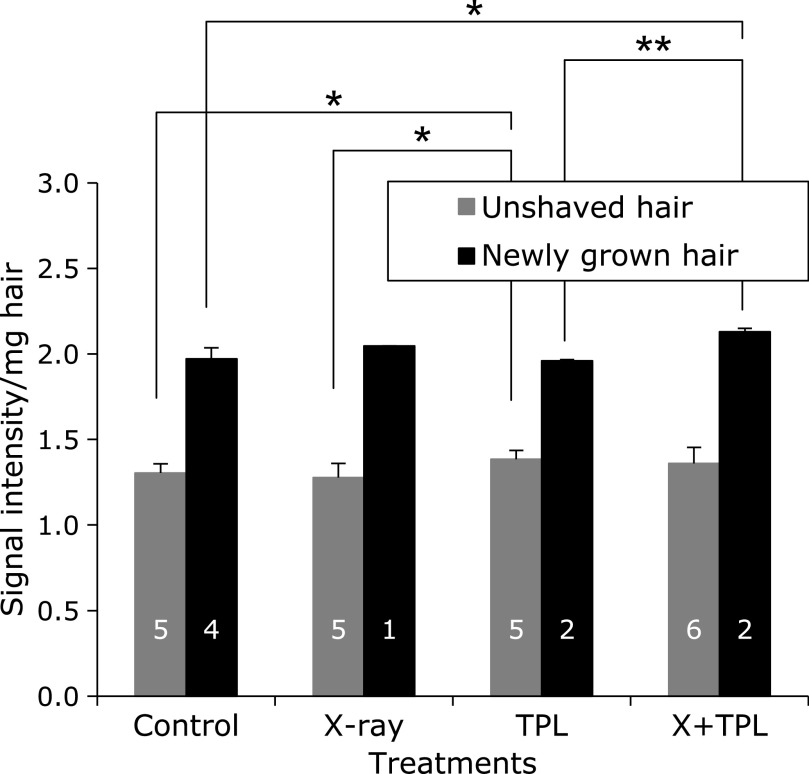
Effects of long-term low-dose (100 mGy/day × 5 days/week × 12 weeks = 6 Gy in total) X-ray irradiation (Protocol 1) on melanin-derived radicals in hair growing on living C3H/He mice. Amounts of melanin-derived radicals in remaining hair (gray column) and the newly grown hair (black column) were compared. Columns and error bars indicate the average ± SD. The numbers indicated on the column are number(s) of mouse/mice available for hair sample preparation. * and ** indicate grades of significance as *p*<0.05 and *p*<0.01, respectively.

The results of Protocol 1, which assessed the effects of long-term low-dose (100 mGy/day × 5 days/week × 12 weeks = 6 Gy in total) X-ray irradiation on melanin-derived radicals in hair on living mice, are shown in Fig. 3. The amount of melanin-derived radicals was significantly increased in the newly grown hair (black column) compared with the remaining hair (gray column). The numbers indicated on the column are number(s) of mouse/mice available for sample preparation. Only one mouse was available in the X-ray irradiated group to collect re-grown hair. However, no differences were found between irradiated and non-irradiated groups, and no effects of TPL in the drinking water were noted. X-ray treatment and/or TPL treatment slightly affected hair regrowth, but their significance was not demonstrated in this single experiment.

**Fig. 4:**
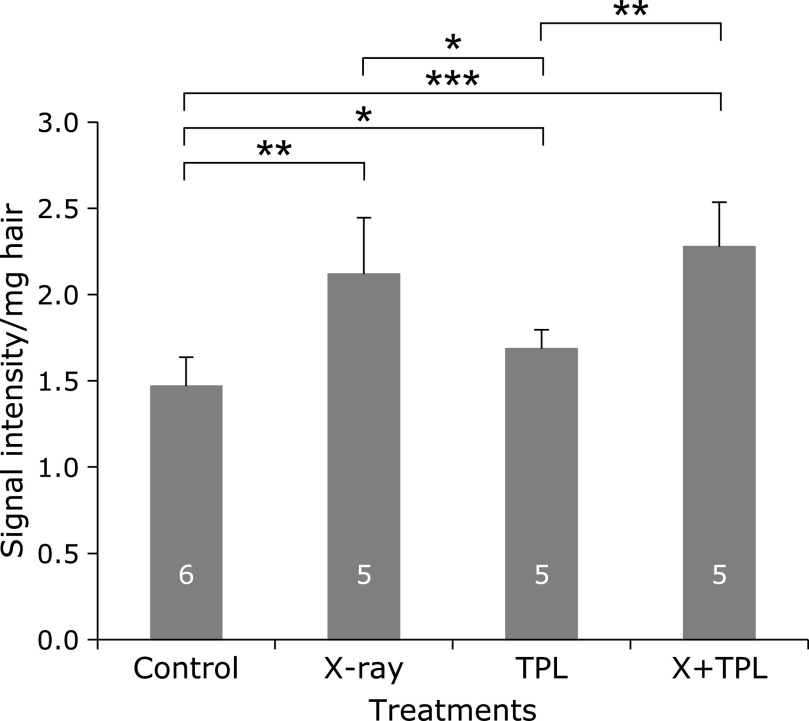
Effects of long-term low-dose (500 mGy/day × 5 days/week × 4 weeks = 10 Gy in total) X-ray irradiation (Protocol 2) on melanin-derived radicals in hair growing on living C3H/He mice. Columns and error bars indicate the average ± SD. The numbers indicated on the column are numbers of mice used for hair sample preparation. *, **, and *** indicate grades of significance as *p*<0.05, *p*<0.01, and *p*<0.001, respectively.

The results of Protocol 2, which assessed the effects of long-term low-dose (500 mGy/day × 5 days/week × 4 weeks = 10 Gy as total) X-ray irradiation on melanin-derived radicals in hair on living mice, are shown in Fig. 4. The amount of melanin-derived radicals in hair sampled after the 4-week irradiation period was higher in the irradiated groups than in the non-irradiated groups. No effects of TPL added in the drinking water were noted. The daily dose of 500 mGy affected the amount of melanin-derived radicals in hair after the 4-week irradiation period. Although 60% of the total dose in Protocol 2 was administered in Protocol 1, the daily dose of 100 mGy during 12 weeks had almost no effect on the amount of melanin-derived radicals.

The amount of melanin-derived radicals in mouse hair increased after 4-week X-ray irradiation at 500 mG/day (Fig. 4). However, the baseline was changed after hair regrowth (Fig. 3). Furthermore, regrown hair was not collected for all mice used in the experiment. The periods until starting regrowth after shaving varied among mice. It was therefore difficult to sample the regrown hair from all mice during the experimental period. This was probably due to the hair cycle. Seasons may also affect hair growth and the amount of melanin-derived radicals in mouse hair. The melanin-derived radicals in mouse hair may respond to radiation exposure, but hair samples may be slightly difficult to use in practice without additional processing.

**Fig. 5:**
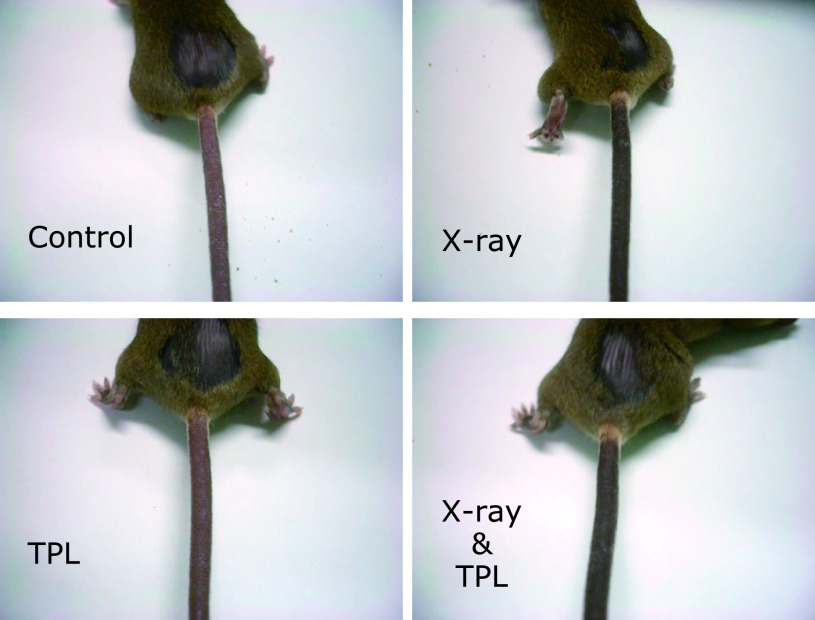
Tanning of tail skin of C3H/He mice by X-ray irradiation during Protocol 2. Images were taken at day-23.

X-ray irradiation increased the amount of melanin in skin, and browning of the ear tip and tail was observed 3 weeks after start of Protocol 2. Images of mouse tails taken at day-23 are shown in Fig. 5. TPL in the drinking water had no effect on tanning of the tail skin by visual assessment.

**Fig. 6:**
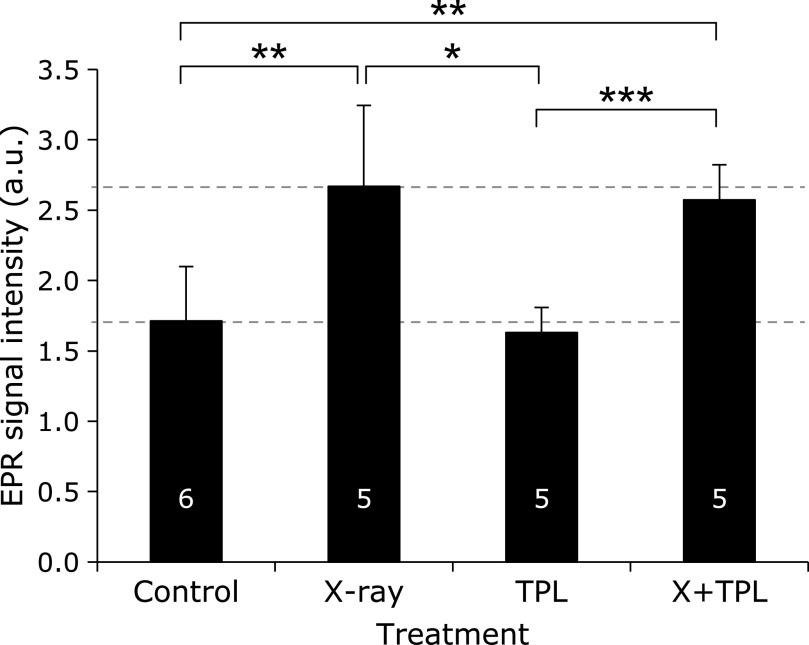
Effects of long-term low-dose (500 mGy/day × 5 days/week × 4 weeks = 10 Gy in total) X-ray irradiation (Protocol 2) on melanin-derived radicals in tail skin of living C3H/He mice. Columns and error bars indicate the average ± SD. The numbers indicated on the column are numbers of mice used for hair sample preparation. *, **, and *** indicate grades of significance as *p*<0.05, *p*<0.01, and *p*<0.001, respectively.

The amount of melanin-derived radicals in tail skin of C3H/HeSlc mice after Protocol 2 is shown in Fig. 6. The amount of melanin-derived radicals significantly increased in X-ray-irradiated groups. The amount of melanin-derived radicals was lower in TPL groups, but not significantly. The ratio of increase in the amount of melanin-derived radicals for 500 mGy/day treatment compared with the control was 1.56 for C3H/HeSlc mice.

**Fig. 7:**
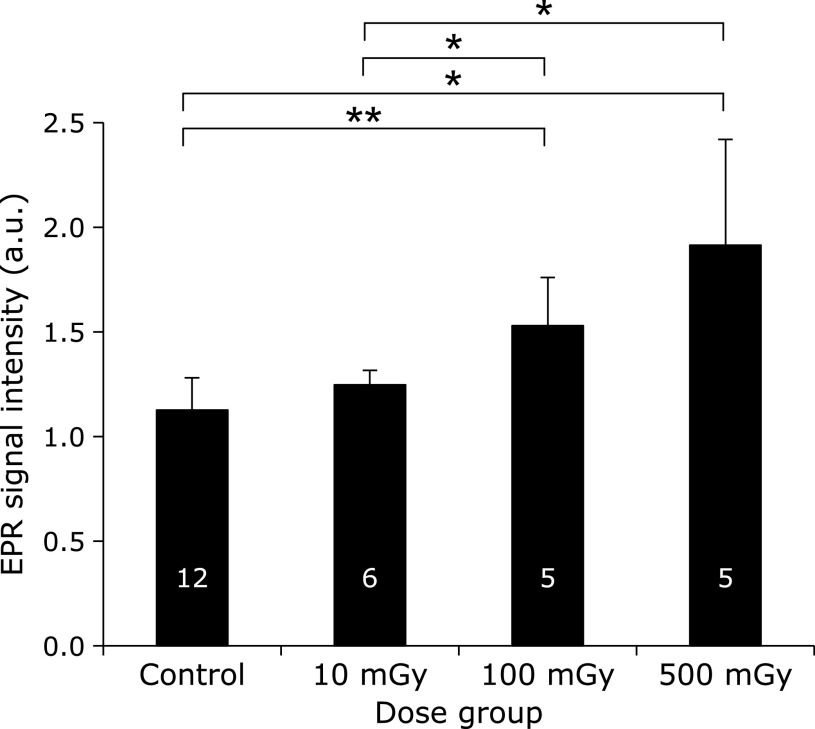
Effects of X-ray dose on the amount of melanin-derived radicals in C57BL/6N Cr mouse tail skin. Mice were irradiated with X-rays at 0, 10, 100, or 500 mGy/day 5 times a week for 4 weeks. The total dose was 0.0, 0.2, 2.0, or 10 Gy, respectively. Columns and error bars indicate the average ± SD. The numbers indicated on the column are numbers of mice used for hair sample preparation. * and ** indicate grades of significance as *p*<0.05 and *p*<0.001, respectively.

The amount of melanin-derived radicals in tail skin of C57BL/6NCrSlc mice after Protocol 3 is shown in Fig. 7. Another mouse strain having melanin was tested. It was previously reported by non-invasive EPR measurement that C57BL/6NCrSlc mice have intrinsic melanin-derived radicals in tail skin, albeit fewer than C3H/HeSlc mice.^(5)^ We investigated whether the response of melanin-derived radicals to X-ray radiation is common among chromatic mouse strains, even though the baseline amount of melanin-derived radicals differs. The amount of melanin-derived radicals in tail skin of C57BL/6NCrSlc mice increased dose dependently. The ratio of increase in melanin-derived radicals for 500 mGy/day treatment compared with the control was 1.70 for C57BL/6NCrSlc mice.

As TPL did not prevent the production of radiation-induced melanin-derived radicals in this study, ROS generated in the skin may not induce or help form the melanin-derived radicals. The intake of TPL was calculated as 3.7 µmol/day/mouse when the water intake was 3.7 ± 0.4 ml/day/mouse. The maximum-tolerated dose (MTD) of TPL by i.v. bolus injection was reported to be 275 mg/kg body weight or 32 µmol/20 g body weight.^(9)^ Therefore almost one-tenth of the MTD of TPL for i.v. injection was ingested daily from drinking water. Single bolus i.v. injection of 0.12 or 0.34 µmol/20 g body weight of Edarabone (Radicut), which is a clinically used cerebral neuroprotective drug with antioxidative function, was reported to suppress anthralin-derived radical generation in the skin.^(10)^ Oral administration of 200 µl/20 g body weight/day of commercialized antioxidative nutritional supplement, which contains several herbal antioxidants 0.1–100 µg/µl, for 3 days also suppressed anthralin-derived radical production in the skin.^(10)^ The dose of TPL in this study was not different from the doses of antioxidants functioning in the skin.

Melanin-derived radicals were not observed in albino mice species such as BALB/cCrSle, Slc:ddY, or Slc:ICR.^(5)^ Similarly, melanin-derived radicals may not be a good index of radio-biological effects for all humans. X-ray irradiation caused browning of the tail and increased amounts of melanin-derived radicals in the skin of brown/black mouse species. Melanin-derived radicals in the skin have the possibility to be an endogenous marker for long-term and low-dose irradiation for species having melanin.

## Conclusions

Exposing mice to several hundred mGy of X-rays daily caused tanning, and increased the production of melanin-derived radicals in hair and tail skin. Melanin-derived radicals in hair may reflect X-ray irradiation, but the baseline amount varies. However, the amount of melanin-derived radicals in the tail skin increased dose dependently. Melanin-derived radicals in skin can be a temporal marker for long-term and low-dose irradiation.
